# Disability and psychological burden in severe obesity: results from a structured psychosomatic assessment of candidates for metabolic and bariatric surgery

**DOI:** 10.3389/fpsyt.2026.1849789

**Published:** 2026-07-08

**Authors:** Angelika Thönnes, Sebastian Holländer, Gudrun Wagenpfeil, Michael Noll-Hussong, Christoph Heinz, Matthias Riemenschneider

**Affiliations:** 1 Department of Psychiatry and Psychotherapy, Saarland University Medical Center (UKS), Homburg, Germany; 2 Psychoanalysis, Psychotherapy and Psychosomatic Medicine, Saarland University, Homburg, Germany; 3 Department of General Surgery, Visceral Surgery, Vascular Surgery and Pediatric Surgery, Saarland University Medical Center (UKS), Homburg, Germany; 4 Institute of Medical Biometry, Epidemiology and Medical Informatics (IMBEI), Saarland University Medical Center (UKS), Homburg, Germany; 5 Psychosomatic Day Clinic Westend, Munich, Germany

**Keywords:** bariatric surgery, depression, disability, obesity, personality functioning, psychosomatic assessment, WHODAS 2.0

## Abstract

**Background:**

Metabolic and bariatric surgery (MBS) is an evidence-based treatment for severe obesity and obesity-related disease. A psychosomatic assessment is widely recommended as part of the preoperative evaluation; however, standardized approaches remain limited. This cross-sectional study evaluated a structured assessment combining detailed interviews, psychometric questionnaires, and measures of psychosocial functioning and disability.

**Patients and methods:**

A total of 220 adults with severe obesity who underwent a structured preoperative psychosomatic assessment during routine evaluation for metabolic and bariatric surgery between 2019 and 2022 at Saarland University Medical Center were included. Patient-reported outcomes were obtained using the PHQ-D (including PHQ-9 and PHQ-15), GAD-7, WHODAS 2.0 (12-item version), AUDIT, OPD-SQS (12-item), and EDE-Q. Therapist-administered ratings included GAF, GARF, and diagnostic assignments.

**Results:**

Of the 220 patients, 69.1% were female and 30.9% male, with a mean age of 41.8 years (SD = 11.6) and a mean BMI of 51.8 kg/m² (SD = 8.4). A prior history of mental illness was reported by 37.7%. Over 60% met DSM-IV Axis I criteria for current psychopathology, predominantly affective (54.5%), anxiety (24.5%), and eating disorders (19%). The mean WHODAS score was 20.3, with 76.7% showing clinically significant disability (≥25%). Emotional distress related to health problems was most frequently endorsed (64.5%). Somatic comorbidities included prediabetes or type 2 diabetes in 31.8%. Moderate-to-severe depressive symptoms were observed in 47.1%. WHODAS scores correlated significantly with PHQ-9 (r = .57, p <.001) and PHQ-15 (r = .58, p <.001) but not with BMI (r = .05, p = .49). The EDE-Q total mean was 3.3 (SD = 1.1), indicating abnormal eating behavior. The OPD-SQS mean was 16.8 (SD = 9.8), with relational difficulties moderately correlated with disability (r = .33, p <.001).

**Conclusion:**

Candidates for MBS showed substantial psychological and functional burden. Disability was more strongly associated with depressive symptoms, somatic distress, and psychosocial functioning than with BMI. These findings support the integration of structured psychosomatic assessment into routine preoperative bariatric evaluation and emphasize the importance of individualized perioperative mental health care.

## 1 Introduction

Due to its high and rising prevalence, along with its associated risks of morbidity and mortality, overweight (BMI 25–29.9 kg/m²) and obesity (BMI ≥30 kg/m²) rank among the most significant global health challenges (1). According to data from the Study of Adult Health in Germany (Robert Koch Institute), approximately 60% of adults in Germany are overweight, with a higher prevalence in men (67.1%) than in women (53%) (2). The prevalence of obesity is approximately 24%, with no significant gender difference (3).

Severe obesity exhibits all the characteristics of a chronic disease, particularly due to its profound impact on functional status, quality of life, and psychological well-being. A significant correlation between psychological symptom severity and increasing BMI has been documented (4). Studies report a high prevalence of depression, anxiety symptoms, and disordered eating behaviors, particularly among patients with a BMI >50 (5). Among individuals referred for bariatric surgery, the prevalence of mental disorders ranges from 38% to 56%, with depression (14–25%, mean 19%), anxiety disorders (6–20%, mean 12%), and binge eating disorder (13–21%, mean 17%) being the most common (6–9). Meta-analyses of prospective studies indicate that obesity increases the likelihood of developing depression (10, 11). This association is likely bidirectional: the burden of living with obesity may heighten the risk of developing subclinical or clinical depression, while depressive symptoms can contribute to weight gain and obesity via lifestyle changes, altered health behaviors, and endocrinological mechanisms (12). Obesity and depression are strongly linked and both are closely associated with disability (13, 14), though few studies have specifically examined this interrelationship (15).

Metabolic and bariatric surgery (MBS) is an evidence-based intervention associated with substantial and sustained weight loss, improvement of obesity-related comorbidities, enhanced quality of life, and reduced long-term mortality in patients with severe obesity (16). According to the 2022 ASMBS/IFSO guidelines, MBS is recommended for individuals with BMI ≥35 kg/m² regardless of comorbidities, and for patients with type 2 diabetes and BMI >30 kg/m²; it should also be considered in selected patients with BMI 30–34.9 kg/m² who do not achieve durable benefit from nonsurgical treatment (17). The integration of a mental health professional (MHP) into the interdisciplinary treatment team is recommended, as psychological comorbidities are more prevalent in patients undergoing bariatric surgery compared to the general population and may negatively influence postoperative adherence, quality of life, and psychosocial recovery if left untreated (6, 8, 18, 19). Patient-centered psychometric tools, such as the Patient Health Questionnaire-D (PHQ-D), have become increasingly prominent in preoperative evaluations (20). The PHQ-9 assesses depressive symptom severity (21), while the PHQ-15 evaluates somatic symptom burden (22). Additionally, the WHO Disability Assessment Schedule 2.0 (WHODAS 2.0, 12-item version) enables the multidimensional quantification of impairments in activity, participation, and function, making it a valuable tool for evaluating preoperative and perioperative outcomes (23).

Previous studies have demonstrated that patients with high BMI and waist circumference experience significantly impaired quality of life (24). The WHODAS 2.0 has been validated as a highly reliable tool for measuring disability in the obese population (15, 25, 26), further underscoring the need for comprehensive psychosomatic assessments in bariatric surgery candidates.

Despite the high prevalence of psychological comorbidities in bariatric surgery candidates, standardized approaches that integrate psychiatric diagnosis, psychosocial functioning, and disability assessment within a single structured evaluation remain limited. In particular, few studies have examined how psychopathology, personality functioning, and disability interact in patients with severe obesity undergoing surgical evaluation. Addressing this gap may improve the clinical relevance of preoperative psychosomatic assessments.

The primary objective of this study was to characterize psychosomatic burden and disability in patients undergoing preoperative evaluation for metabolic and bariatric surgery using a structured multidimensional assessment approach.

Secondary objectives were (1): to examine associations between disability and psychological symptom burden (2), to evaluate the relationship between BMI and psychosocial functioning, and (3) to explore associations between personality functioning and functional impairment.

The assessment approach intentionally combined categorical diagnostic frameworks (ICD-10 and DSM-oriented symptom measures) with dimensional psychometric instruments assessing disability, psychosocial functioning, and personality structure. This integrated biopsychosocial approach was selected to capture clinically relevant aspects of psychological burden that may not be sufficiently represented by categorical psychiatric diagnoses alone.

## 2 Materials and methods

### 2.1 Participants

A total of 220 adult patients with severe obesity (69.1% female, 30.9% male) with a mean age of 41.8 years (SD = 11.6) and an average BMI of 51.8 kg/m² (SD = 8.4) were evaluated preoperatively at Saarland University Medical Center (UKS). Patients were consecutively referred for psychosomatic preoperative evaluation as part of the standard interdisciplinary bariatric surgery pathway at Saarland University Medical Center between 2019 and 2022.

The present study employed a cross-sectional observational design using a single preoperative assessment time point. The assessment focused on psychosomatic and psychosocial factors in accordance with the S3 guidelines issued by professional societies, including the German Obesity Society (DAG) (27) and the guidelines for Surgery of Obesity and Metabolic Diseases (16). This study was reviewed and approved by the Ethics Committee of the Saarland Medical Association.

### 2.2 Procedure

Before undergoing psychosomatic evaluation, indication and surgical eligibility had already been assessed from a surgical perspective. Patients exhibiting marked affective instability and impaired behavioral self-regulation interfering with perioperative adherence, acute suicidality, active substance use disorder, uncontrolled bulimia nervosa were excluded, as these are considered contraindications for bariatric surgery.

All assessments were conducted at a single preoperative time point during routine psychosomatic evaluation prior to final surgical approval.

The mental health evaluation was conducted by a specialized psychotherapist with expertise in obesity treatment. To improve standardization, assessments followed a structured interview framework routinely used in the bariatric psychosomatic outpatient clinic. Diagnostic assignments were subsequently reviewed by an experienced senior clinician according to ICD-10 criteria.

The assessment consisted of a structured clinical interview evaluating eating behavior, mood regulation, alcohol and drug use, history of stressful experiences or trauma, social support system, as well as clinician-administered and self-report questionnaires to assess disability and personality functioning.

Most self-report questionnaires were distributed during a previous outpatient appointment and returned completed at the time of psychosomatic evaluation, whereas clinician-administered ratings (e.g., GAF, GARF) were completed during the interview session.

### 2.3 Materials

ICD-10 criteria were used for formal clinical diagnoses, whereas DSM-oriented dimensional instruments (e.g., PHQ-9, GAD-7, WHODAS 2.0) were employed to quantify symptom severity and functional impairment across relevant psychosocial domains.

The following questionnaires were used for patient-reported parameters: Patient Health Questionnaire-D (PHQ-D), includes the Patient Health Questionnaire-9 (PHQ-9) to assess the severity of depressive symptoms (20, 21). The PHQ-9 is a self-rating scale consisting of 9 items each rated from 0 to 3, with higher scores indicating greater severity. These items correspond to the nine diagnostic criteria for major depressive episode in the DSM-IV (1994), covering: anhedonia, depressed mood, sleep disturbances, fatigue, appetite changes, low self-esteem/guilt, poor concentration, psychomotor agitation or retardation, and thoughts of self-harm (21). Score interpretation: 0-4: minimal or no depression; 5–9, mild depression; 10–14, moderate depression; 15–19, moderately severe depression; and 20–27, severe depression (21). The Patient Health Questionnaire-15 (PHQ-15) to measure somatic symptoms severity (22), the GAD-7 based on the DSM-5 diagnostic criteria assessing generalized anxiety disorder symptoms (28). The WHO Disability Assessment Schedule 2.0 (WHODAS 2.0, 12-item questionnaire) provides a multidimensional quantification of impairments in activity, participation, and function (23, 29). The Alcohol Use Disorders Identification Test (AUDIT) screening for alcohol-related problems (30, 31), the Operationalized Psychodynamic Diagnostic (OPD-SQS) for evaluation of psycho-structural pathology (32) and the Eating Disorder Examination-Questionnaire (EDE-Q) (33) to assess eating disorder-related behaviors and cognitions. In terms of therapist-administered assessment scales, the following were used: the Global Assessment of Functioning (GAF) evaluates overall psychosocial functioning (34), the Global Assessment of Relational Functioning (GARF) (35) assesses relational and interpersonal functioning and F-Diagnosis, respectively Z-Diagnosis (ICD-10) for diagnostic purposes to distinguish between clinical disease states and psychosocial distress.

Most self-report questionnaires were provided to patients during an earlier outpatient appointment and returned completed at the time of evaluation.

Additional somatic parameters were recorded, including: comorbid conditions with a focus on Type 2 Diabetes mellitus and Prediabetes. Anthropometric measurements assessed were height, weight, waist circumference and body mass index (BMI). Z codes were established in ICD-10 to clarify the distinction between “disease” and “states of suffering” due to complaints or stress.

### 2.4 Statistical methods

Categorical variables are presented as absolute and relative frequencies (%). Continuous variables are reported as mean ± standard deviation (SD) or mean and range, depending on the context. Normality of data distribution was assessed using the Shapiro–Wilk test and visual inspection of Q–Q plots before conducting group comparisons.

The t-test and ANOVA were used to compare WHODAS 2.0 scores between groups. For BMI comparisons, the Kruskal-Wallis test was applied due to its suitability for non-normally distributed data. Correlations between variables were analyzed using Spearman’s rank correlation coefficient. A p-value of 0.05 was considered statistically significant. All statistical analyses were conducted using IBM SPSS Statistics version 28. The analyses were primarily exploratory and descriptive in nature and aimed to characterize multidimensional psychosomatic burden in a clinically complex bariatric population. Future studies with longitudinal designs and larger multicenter samples should apply multivariate modeling approaches to identify independent predictors of disability, psychosocial functioning, and postoperative outcomes.

## 3 Results

### 3.1 Sociodemographic findings

The demographic characteristics of the sample including age, gender, BMI, marital status, and education level are summarized in **Table 1**. Among the 220 patients with severe obesity, the majority were female (n = 152, 69.1%) while 30.9% (n = 68) were male. The mean age was 41.8 years (range 18–72) with women averaging 41.3 years and men 43.0 years. The mean BMI was 51.8 kg/m² (range 36–85 kg/m²), with men averaging 52.4 kg/m² and women 51.4 kg/m².

**Table 1:** Descriptive characteristics of the study population.

Characteristic	Total (N = 220)	Male (n=68)	Female (n=152)
Age, years	41.8 (11.6)	43.0 (11.5)	41.3 (11.6)
BMI, kg/m²	51.8 (8.4)	52.4 (8.1)	51.4 (8.5)
Waist circumference			
< 130 cm	18 (11.4%)	0	18 (15.9%)
130–159 cm	111 (70.3%)	28 (62.2%)	83 (73.5%)
≥160 cm	29 (18.4%)	17 (37.8%)	12 (10.6%)
Education level			
Primary school diploma	106 (49.5%)	39 (60%)	67 (48%)
Middle school diploma	79 (36.9%)	19 (29.2%)	60 (40.3%)
High school diploma	29 (13.6%)	7 (10.8%)	22 (14.8%)
Marital status			
Single	49 (22.7%)	22 (32.4%)	27 (18.2%)
Single with child	39 (18.1%)	5 (7.4%)	34 (23.0%)
Married, solid partnership	128 (59.3%)	41 (60.3%)	87 (58.82%)
Employment status			
Health-related incapacity for work	25 (11.6%)	9 (13.6%)	16 (10.7%)
Retired	15 (7.0%)	5 (7.6%)	10 (6.7%)
Employed full-time	75 (34.9%)	28 (42.4%)	47 (31.5%)
Employed part-time	38 (17.7%)	6 (9.1%)	32 (21.5%)
Unemployed	62 (28.8%)	18 (27.3%)	44 (29.5%)

Values are mean (SD) or n (%). Percentages are based on valid cases.

Regarding education level, 42.1% of patients had a low level of education, 28.8% were unemployed, and 11.6% reported an occupational disability due to health-related issues (see **Table 1**).

### 3.2 Exclusion criteria

Following evaluation, 2.8% (n = 6) of patients were excluded due to a marked affective instability, including severe alcohol dependence (1.4%; n = 3) and uncontrolled bulimia nervosa (1.4%; n = 3), both considered contraindications according to current guidelines. Additionally, 16.4% (n = 36) of patients required further specialist psychiatric and/or psychotherapeutic treatment before proceeding with surgery. For these individuals, a follow-up assessment was scheduled after six months to ensure sufficient psychological and psychosocial stability for the postoperative phase.

### 3.3 Anthropometric and comorbid findings

In addition to a mean BMI of 51.8 kg/m² all patients exhibited significantly high waist circumference measurements with 70.3% falling between 130–159 cm. Furthermore, 31.8% of patients had a pre-existing diagnosis of pre-diabetes or type 2 diabetes mellitus (NIDDM or IDDM), with a slightly higher prevalence among men (35.3% male, 30.3% female).

### 3.4 Previous mental illness

A history of mental illness was reported in 37.7% (n = 83) of patients with 18.4% having received outpatient treatment, 2.3% inpatient treatment only, and 17.5% undergoing both outpatient and inpatient psychiatric or psychotherapeutic treatment either in the past or currently. A total of 3.2% (n = 7) reported previous suicide attempts. A history of substance addiction was documented in 33.6% of patients with nicotine abuse being the most prevalent (28.2%).

### 3.5 Psychiatric diagnoses after evaluation: F-diagnoses

Following a comprehensive evaluation, 79.1% of patients (174 out of 220) were diagnosed with at least one F diagnosis, with an average of 1.3 F diagnoses per patient. Gender-related differences were observed across all categories, except for eating disorders (See **Table 2**).

**Table 2:** Lifetime prevalence of mood, panic, eating and addictive disorders.

Diagnosis	Total (N = 220)	Male (n=68)	Female (n=152)
Any psychiatric disorder (Multiple answers possible)	174 (79.1%)	52 (76.5%)	122 (80.3%)
Mental and behavioral disorder due to drug use	40 (18.2%)	20 (29.4%)	20 (13.2%)
alcohol addiction	3 (1.4%)	2 (2.9%)	0
nicotine abuse	35 (15.9%)	20 (29.4%)	15 (9.9%)
other drug abuse	2 (0.9%)	0	2 (1.3%)
Schizophrenia	2 (0.9%)	0	2 (1.3%)
Affective disorders	120 (54.5%)	32 (47.1%)	88 (57.9%)
mild depression F32.0, F33.0	74 (33.6%)	32 (47.1%)	42 (27.6%)
major depression F32.2, F33.2	46 (20.9%)	0	46 (30.3%)
Anxiety disorder, neurotic-, stress-related and somatoform disorders	54 (24.5%)	19 (27.9%)	35 (23.0%)
Eating disorders	42 (19.1%)	13 (19.1%)	29 (19.1%)
Personality disorders	12 (5.5%)	5 (7.4%)	7 (4.6%)
Behavioral and emotional disorders with onset occurring in childhood, adolescence	10 (4.5%)	7 (10.3%)	3 (2.0%)

### 3.6 Z-diagnoses

In most areas, except for lifestyle-related issues, the burden measured by Z-Diagnoses was more prevalent among women (See **Table 3**). Z-Diagnoses are used for documenting reasons for healthcare encounters that are not directly related to a disease or injury.

**Table 3:** Z-diagnoses.

Z-Diagnosis	Total (N = 220)	Male (n=68)	Female (n=152)
Problems related to work	85 (38.6%)	24 (35.3%)	61 (40.1%)
Problems related to social environment, Stigmatization	35 (15.9%)	8 (11.8%)	27 (17.8%)
Negative events in childhood	45 (20.5%)	11 (16.2%)	34 (22.4%)
Problems related to primary support group	76 (34.5%)	18 (26.5%)	58 (38.2%)
Problems related to lifestyle	178 (80.9%)	59 (86.8%)	119 (78.3%)
Accentuation of personality traits	60 (27.3%)	17 (25.0%)	43 (28.3%)
Others	27 (12.3%)	7 (10.3%)	19 (12.5%)

Z codes *(Z00 – Z99)* of the ICD-10-CM coding manual indicate a reason for an encounter or provide additional information about a patient.

### 3.7 Psychometric findings

An overview of the psychometric results is presented in **Table 4**.

**Table 4:** Psychometric findings.

Measure	N	Mean	Standard deviation	Minimum	Maximum
PHQ-9	220	10	4.9	2	24
PHQ-15	220	11.4	5	1	25
WHO-DAS	219	20.3	9.9	0	42
GAF	220	67.1	12	45	90
GARF	220	73.4	10.7	30	90
OPD-SQS	153	16.8	9.8	1	44
EDE-Q	219	3.3	1.1	0.6	5.8

#### 3.7.1 Patient health questionnaire

The average PHQ-9 score was 10 (SD = 4.9), indicating distinct symptoms of depression compared to the general population (36), where the mean PHQ-9 score for the specific age group of 45–54 years is 2.8 (SD = 3.5). Additionally, the average PHQ-15 score was 11.4 (SD = 5.0), suggesting moderate to severe levels of somatization. Depressive disorder was assessed using the nine-item Patient Health Questionnaire (PHQ-9), with a score of 10 or higher defining clinically significant depression. 47.1% of patients exhibited clinically significant depressive symptoms, with a higher prevalence in women; however, no statistically significant gender differences were found in the PHQ-9 categories. Moderately severe or severe somatic symptoms were reported by 75.9% of patients (69.2% male, 78.9% female). While women scored higher in the PHQ-15 severe symptom category, no significant gender differences were observed across PHQ-15 classifications (See **Table 5**).

**Table 5:** PHQ-9 and PHQ-15 grouped by severity of symptoms.

Measure	Total, n (%)	Male, n (%)	Female, n (%)
PHQ-9 groups			
No depression 0–4	37 (16.8%)	10 (14.7%)	27 (17.8%)
Mild D. 5–9	79 (35.9%)	26 (38.2%)	53 (34.9%)
Moderate D. 10–14	61 (27.7%)	21 (30.9%)	40 (26.3%)
Moderately severe to severe D. ≥15	43 (19.5%)	11 (16.2%)	32 (21.1%)
PHQ-15 groups			
None-minimal somatic symptoms 0–4	10 (4.5%)	4 (5.9%)	6 (3.9%)
Low somatic symptoms 5–9	43 (19.5%)	17 (25.0%)	26 (17.1%)
Medium somatic symptoms 10–14	93 (42.3%)	32 (47.1%)	61 (40.1%)
High somatic symptoms 15–30	74 (33.6%)	15 (22.1%)	59 (38.8%)

PHQ-9, PHQ-15: Depression severity was categorized based on the PHQ-9 using the following cutoffs: minimal (0–4), mild (5–9), moderate (10–14), and moderately severe to severe (≥15), with the original categories “moderately severe” and “severe” combined. Somatic symptom severity was classified based on the PHQ-15 using the following cutoffs: minimal (0–4), low (5–9), medium (10–14), and high (≥15).

#### 3.7.2 World health organization disability assessment schedule 2.0

The WHODAS 2.0 score, a functional measure aligned with the DSM-5, was 20.3 (42.3% of the maximum possible score) with a range from 0 to 42, indicating substantial disability burden in the sample. A total of 168 patients (76.7%) had a WHODAS score of ≥25%, with no significant gender differences.

Marked difficulties were reported in the following items: S5 “Emotional impairment due to the health disorder” (64.5%), S1 “Standing for 30 min” (51.9%), S7 “Walking about 1 km” (56.5%), S4 “Participation in social activities” (40.9%).

Additionally, WHODAS scores differed significantly between PHQ-9 and PHQ-15 severity categories (ANOVA, p <.001).

No significant association was observed between WHODAS scores and BMI, nor between WHODAS scores and gender. Item S1 (‘Standing for 30 minutes’) showed a moderate association with BMI (p = .004), whereas no significant associations were observed for emotional impairment or participation in social activities (See **Table 6**).

**Table 6:** WHODAS 2.0 distribution of mean scores by age group, sex and BMI group.

Variable	Patients with clinically significant disability (%)	Mean WHODAS score (SD)	Mean score as % of maximum	Score range	P-value
Total (n=219)	168 (76.7%)	20.3 (9.9)	42.3%	0–42	
Sex					
Male (n=67)	51 (76.1%)	20.3 (10.4)	42.3%	1–42	0.96
Female (n=152)	117 (77.0%)	20.3 (9.7)	42.3%	0–42	
BMI category					
BMI 37.5–47.3 (n=68)	52 (76.5%)	20.1 (9.4)	41.8%	2–41	0.72
BMI 47.4–53.1 (n=72)	48 (66.7%)	19.7 (12.0)	41.0%	0–42	
BMI > 53.1 (n=79)	68 (86.1%)	21.0 (8.1)	43.8%	5–40	
Age group					
Age < 41 years (n=114)	82 (71.9%)	19.8 (9.9)	41.3%	0–40	0.08
Age ≥ 41 years (n=105)	86 (81.9%)	21.5 (9.7)	44.8%	1–42	

SD, Standard Deviation.

#### 3.7.3 Eating disorder examination questionnaire

The Eating Disorder Examination Questionnaire (EDE-Q) revealed an elevated mean total score of 3.3 (SD = 1.1), with women scoring 3.4 (SD = 1.2) and men 3.0 (SD = 1.0). This exceeds the recommended cutoff of 2.8 for screening eating disorders and indicates a high prevalence of non-normative eating behaviors in this patient population (37).

#### 3.7.4 Global assessment of functioning

The average GAF score was 67.1 (SD = 12.0, range 45–90), placing most patients in the 61–70 category, which indicates mild symptoms or some difficulty in functioning. The distribution across categories was as follows: 28.6% scored between 51–60, indicating moderate difficulties; 8.6% scored between 41–50, reflecting serious difficulties; 28.2% fell within the 61–70 range, showing mild symptoms; 19.5% scored between 71–80, indicating temporary difficulties. 15% scored 81 or above, reflecting minimal or no difficulties.

#### 3.7.5 Global assessment of relational functioning

The average GARF score was 73.4 (SD = 10.7, range 30–90) with the following distribution: 65.0% scored between 62–80, corresponding to temporarily unsatisfactory relationship patterns; 21.8% scored between 81–100, indicating satisfactory relationship patterns; 12.7% scored between 41–60, denoting mostly unsatisfactory relationship patterns; 0.5% fell within the 21–40 range, suggesting seriously dysfunctional relationship patterns.

#### 3.7.6 The alcohol use disorder identification test

The AUDIT identified risky or hazardous alcohol consumption (scores ≥4 for women and ≥5 for men) in 5.5% of patients (38, 39).

#### 3.7.7 The OPD structure questionnaire short scale

The mean OPD-SQS total score was 16.8 (SD = 9.8, range 0–44), with women scoring higher (17.5, SD = 10.5) compared to men (15.0, SD = 7.6). The most pronounced impairments were observed in the domain of relationship difficulties, including disturbed communication skills, reduced bonding ability, and insecure attachment styles, which were moderately more pronounced in women (male = 6.6, female = 7.9). Distribution of OPD-SQS scores: 46.5% scored between 10–20; 21.6% had low scores (<10); 26.8% scored above 20; 5.1% were in the upper extreme (>30), indicating severe impairments in personality functioning.

The evaluation identified a significant correlation between the OPD-SQS total score and the PHQ-9 (*r*_s_ = .47, p <.001), indicating a strong association between personality functioning and depressive symptoms. Further moderate correlations were observed with: PHQ-15 (*r*_s_ = .30, p <.001), suggesting a link between personality functioning and somatic symptom severity. WHODAS (*r*_s_ = .35, p <.001), reflecting an association with impairments in activities and participation. EDE-Q (*r*_s_ = .29, p <.001), particularly in the Eating Concern subscale (*r*_s_ = .35, p < 0.001), indicating a relationship between personality structure and eating disorder-related concerns.

Conversely, significant inverse correlations were found with: GAF (*r*_s_ = -.60, p <.001), suggesting that lower personality functioning is linked to greater overall impairment. GARF (*r*_s_ = -.64, p <.001), indicating a negative association between personality structure and relational functioning.

#### 3.7.8 Supplementary insights

No significant correlations were observed between OPD-SQS total scores and BMI. Similarly, BMI was not significantly associated with depressive symptom severity (PHQ-9) or overall functional impairment (WHODAS). Pre-diabetes or Type 2 diabetes was documented in 31.8% of patients. While mean scores for depressive (PHQ-9: 10.7 vs. 9.6) and somatic symptoms (PHQ-15: 13.5 vs. 12.6) were higher in patients with diabetes, the differences were not statistically significant.

## 4 Discussion

This study provides a comprehensive characterization of patients seeking metabolic and bariatric surgery, revealing substantial comorbidities, functional impairments, and disabilities that may not be fully captured in routine preoperative assessments. More than 60% of patients met DSM-IV Axis I criteria for comorbid psychopathology, with the most prevalent conditions being: affective disorder (54.5%), anxiety and stress related disorder (24.5%) and eating disorder (19%).

Additionally, a high level of preoperative disability was observed, with 76.7% of patients scoring ≥25% on the WHODAS. While disability scores correlated significantly with depressive symptoms (p <.001), BMI was not associated with disability levels, despite expectations to the contrary.

Our study’s sociodemographic data align with previous research on patients with metabolic and bariatric surgery (5), including the prevalence of major depression (20.9%), which is comparable to findings by Dawes et al. (2016) (19% depression; 95% CI: 14%-25%) (6).

Using the PHQ-9, we found that 47.1% of patients exhibited moderate to severe depressive symptoms, with no significant gender differences. For the final psychiatric diagnosis, results from the semi-structured interview were reviewed by a clinical expert, and ICD-10-based diagnoses were assigned. However, depression prevalence estimates in patients with metabolic and bariatric surgery vary across studies, largely due to differences in assessment tools and cut-off values. Levis et al. (2019) (35) BMJ highlight these inconsistencies, while Cassin et al. (2012) propose a PHQ-9 cutoff of 15 (instead of 10) for patients with metabolic and bariatric surgery to reduce overdiagnosis, given that obesity itself often involves somatic symptoms (e.g., fatigue, inactivity, and disordered eating) that overlap with depressive criteria (40). Additionally, some studies suggest that preoperative psychological evaluations may underestimate psychiatric comorbidities due to patient concerns about surgery eligibility (41). This potential underreporting bias underscores the need for structured diagnostic approaches to accurately assess mental health conditions in this population.

In our study, BMI was not significantly associated with depressive symptom severity, somatic symptom burden, or overall WHODAS 2.0 disability scores. These findings may indicate that disability in severe obesity is influenced by additional psychosocial and functional factors beyond BMI alone. This is consistent with previous evidence suggesting that the relationship between BMI and health-related quality of life may not be strictly linear (42). Our findings support the hypothesis that psychosocial variables may contribute substantially to disability in patients with severe obesity. Due to the cross-sectional design, causal relationships between psychopathology, disability, and obesity severity cannot be inferred. To our knowledge, no published study has failed to find similar associations between common psychopathology and occupational functioning, including recent findings by Ehrenthal et al. (32). This finding further underscores personality functioning as assessed by the OPD-SQS as a significant predictor of psychological distress. Our findings suggest that psychopathology was strongly associated with the extent of individual disability, a pattern that is also reflected in OPD-SQS scores.

Emerging evidence suggests that psychological traits, emotional regulation capacities, and personality functioning may significantly influence postoperative adherence, eating behavior, psychosocial adjustment, and long-term bariatric outcomes. In particular, impairments in emotional regulation and interpersonal functioning have been associated with maladaptive eating patterns and reduced postoperative quality of life (43).

Recent neuropsychological approaches, including neurofeedback-based interventions, have additionally shown promising preliminary effects on emotional eating behavior and body image regulation in post-bariatric populations (44). Structured postoperative psychological follow-up, including interventions targeting emotional regulation, self-efficacy, eating behavior, and cognitive functioning, may contribute to improved postoperative adjustment; however, larger studies with longer follow-up periods are needed to confirm effects on long-term outcomes (19, 43, 44).

Psychosocial factors play a crucial role in chronic conditions such as type 2 diabetes mellitus and obesity. While the negative impact of depression on these diseases is well-documented, the role of stable, personality-oriented factors remains less explored. Further research is needed to determine whether depression and personality functioning in patients with obesity significantly influence the course and severity of type 2 diabetes.

Regarding alcohol use, 2.0% of patients reached an AUDIT total score of ≥8, while 5.5% screened positive when sex-specific thresholds for risky alcohol consumption were applied. Thus, alcohol-related risk in our sample ranged from 2.0% to 5.5%, depending on the applied threshold. These rates are lower than those reported in other bariatric cohorts, such as the preoperative alcohol use disorder frequency of 7.6% reported by King et al. (8). Similarly, other studies have noted a lower reported rate of addictive disorders among candidates with metabolic and bariatric surgery, possibly reflecting a selection effect in the approval process for surgery (45, 46). Additionally, it remains possible that patients underreport psychiatric symptoms, including alcohol use, depression, and stress, due to concerns about surgical approval.

The prevalence of binge-eating disorder (BED) in populations undergoing metabolic and bariatric surgery typically ranges from 15–30% (47), placing the 19% rate of eating disorders observed in this study within the expected range. However, current classification systems may lack the nuance needed to fully capture the spectrum of disordered eating behaviors in obese patients. Notably, Binge-Eating Disorder (BED) is currently classified under F50.8 in the DSM-5, requiring at least one binge episode per week for more than three months, accompanied by significant psychological distress. The ICD-11 includes a distinct diagnosis for BED, reflecting its clinical significance. Beyond BED, obesity-specific, non-normative eating patterns include overeating, “frustration eating,” snacking (“nibbling”), grazing, “night eating syndrome,” cravings, and partial binge-eating disorder. The role of weight concerns in the development of full and partial eating disorders has been extensively documented (48, 49).

The WHODAS 2.0 may serve as a valuable tool for assessing disability across multiple domains, helping both patients and healthcare providers set realistic expectations before bariatric surgery.

From a clinical perspective, these findings suggest that preoperative bariatric assessment should extend beyond BMI and somatic comorbidities and systematically include psychological and functional disability measures. Integrating structured psychosomatic evaluation may help identify patients who could benefit from targeted psychological interventions before and after surgery.

This study aimed to evaluate whether a structured clinical interview, standardized psychometric questionnaires, and psychosocial functioning assessments provide a comprehensive yet practical evaluation of the overall psychosomatic burden in patients with severe obesity prior to surgery.

Our findings suggest that the biopsychosocial stressors affecting patients with severe obesity can be systematically assessed during pre-bariatric evaluations with reasonable effort. When considered together, these factors reveal significant and widespread functional impairments.

The randomized controlled Bariatric Surgery and Education (BaSE) Study demonstrated that psychoeducational follow-up after surgery effectively reduces depressive symptoms and enhances patient self-efficacy. Postoperative psychotherapeutic interventions should be tailored to patient needs and include a specific focus on disordered eating behaviors (19).

These findings should be interpreted in light of the study’s strengths and limitations. Several strengths of this study should be highlighted. First, the assessment combined structured clinical interviews with multiple validated psychometric instruments, allowing a multidimensional evaluation of psychosomatic burden. Second, the relatively large sample of patients with extreme obesity (mean BMI >50 kg/m²) provides clinically relevant insights into a population that remains understudied. Third, the inclusion of functional disability measures (WHODAS 2.0) alongside psychiatric assessment enabled a more comprehensive understanding of patient functioning prior to metabolic and bariatric surgery.

This study also has several limitations. First, the cross-sectional design limits conclusions regarding causal relationships between psychological variables and disability. Second, the study was conducted at a single tertiary bariatric center, which may limit the generalizability of the findings to other clinical settings. Third, self-reporting bias represents an important consideration in the preoperative bariatric setting. Patients may consciously or unconsciously minimize psychological symptoms, maladaptive eating behaviors, or substance use because of concerns that disclosure could negatively affect surgical eligibility. This potential underreporting may contribute to conservative prevalence estimates for psychiatric comorbidity and addictive behaviors.

Because the primary aim of this study was exploratory characterization, no multivariate regression models were applied. Future research should investigate independent predictors of disability and postoperative outcomes using multivariable longitudinal approaches. Due to the exploratory nature of the analyses, no correction for multiple testing was applied, and the results should therefore be interpreted with caution.

## 5 Conclusion

This study demonstrated substantial psychological and functional burden among candidates for metabolic and bariatric surgery. Disability was more strongly associated with depressive symptoms, somatic distress, and psychosocial functioning than with BMI. These findings support the integration of structured psychosomatic assessment into routine preoperative bariatric evaluation and emphasize the importance of individualized perioperative mental health care.

## Data Availability

The original contributions presented in the study are included in the article/supplementary material. Further inquiries can be directed to the corresponding authors.
